# Association Between Aggressive Clinicopathologic Features of Papillary Thyroid Carcinoma and Body Mass Index: A Systematic Review and Meta-Analysis

**DOI:** 10.3389/fendo.2021.692879

**Published:** 2021-06-30

**Authors:** Aliki Economides, Konstantinos Giannakou, Ioannis Mamais, Panayiotis A. Economides, Panagiotis Papageorgis

**Affiliations:** ^1^ Department of Health Sciences, School of Sciences, European University Cyprus, Nicosia, Cyprus; ^2^ Thyroid & Endocrinology Center, Nicosia, Cyprus; ^3^ School of Medicine, European University Cyprus, Nicosia, Cyprus; ^4^ Department of Life Sciences, School of Sciences, European University Cyprus, Nicosia, Cyprus

**Keywords:** papillary thyroid carcinoma, body mass index, BMI, clinicopathologic features, meta-analysis

## Abstract

**Background:**

The association between adiposity and papillary thyroid carcinoma (PTC) has been reported in several studies, but its association with aggressive clinicopathologic features is not well-recognized. Our aim is to systematically review the literature to identify whether adiposity, expressed through Body Mass Index (BMI), is related to aggressive clinicopathologic features such as tumor-node-metastasis (TNM) stage, extrathyroidal extension (ETE), lymph node (LN) metastasis and multifocality in patients with PTC.

**Methods:**

A systematic search for articles was performed using the PubMed, EBSCO, and Cochrane Library for all articles published in English until December 2020. Specific keywords such as “papillary thyroid carcinoma”, “Body Mass Index”, “clinicopathologic features” were used in the search strategy. Two independent reviewers screened all retrieved articles based on predefined inclusion and exclusion criteria. Meta-analysis was performed in the studies that reported crude and adjusted odds ratios (OR). The methodological quality was assessed using the Newcastle-Ottawa Scale.

**Results:**

A total of 11 retrospective cohort studies involving 26,196 participants included. Our findings showed that elevated BMI was significantly associated with ETE in both overweight (OR 1.26, 95% CI: 1.09-1.44) and obesity group (OR 1.45, 95% CI:1.26-1.64). Elevated BMI was also significantly associated with multifocality in overweight patients (OR 1.17, 95% CI:1.10-1.24) and obese patients (OR 1.45, 95% CI:1.29-1.62). Also, obesity was significantly associated with increased tumor size (OR 1.77, 95% CI:1.52-2.03) and with LN metastasis (OR 1.28, 95% CI: 1.12-1.44), whereas being overweight was significantly associated with advanced TNM stage (OR 1.55, 95% CI:1.27-1.83)

**Conclusion:**

Our results provide strong evidence for the association between higher BMI and ETE, multifocality, and tumor size. Further studies with a larger number of participants are required to elucidate further the association of increased BMI with advanced TNM stage and LN metastasis.

## Introduction

The prevalence of thyroid carcinoma has significantly increased worldwide over the past decades, with the highest percentage of increase attributed to a rise in the incidence of papillary thyroid carcinoma (PTC) and predominantly small PTC (1–3). Thyroid cancer incidence has tripled in the United States (US) in the past three decades, with approximately a 3% increase annually from 1974 to 2013 (4). PTC has the fastest increase rate of all malignancies (5). According to the American Cancer Society’s 2020 estimates for thyroid cancer, there are 52,890 new cases, 12,720 in men and 40,170 cases in women (2). This increase may be partially attributed to the more widespread use of high-resolution ultrasonography and fine-needle aspiration biopsy, resulting in the increased diagnosis of smaller tumours (6–8). However, the increase in the detection of more invasive thyroid tumours and the increase in large-sized tumors suggest that additional underlying causes may be responsible for this phenomenon.

Several environmental factors and eating habits, such as iodine intake, cruciferous vegetable consumption, fish consumption, obesity, radiation exposure, imaging with iodine-containing media, and endocrine disruptors, have been implicated in the pathogenesis of thyroid cancer increase (9, 10). Obesity prevalence has also increased worldwide (11). It has tripled in men and doubled in women between 1975 to 2014, according to a study including 19,2 million participants in 200 countries (12). Elevated Body Mass Index (BMI) has been linked to an increased incidence of several cancers, including thyroid (13, 14). Higher BMI has also been associated with more aggressive tumours and higher mortality in various kinds of malignant neoplasms (13, 15, 16). Moreover, 20% of all cancers are associated with excess body weight, making obesity a significant public health challenge and potentially avoidable cause of mortality and morbidity (17).

A definitive association of adiposity and the development of thyroid cancer has not been well established yet. Still, epidemiological data are pointing to an independent association between obesity and increased incidence of thyroid neoplasia. A meta-analysis including 12,199 thyroid cases reported a 25% greater risk of thyroid cancer in overweight patients and a 55% increase in thyroid cancer risk among obese patients when compared with normal weighted individuals (18). Various studies reported a positive association between BMI and thyroid cancer in both men and women, although the relationship between obesity and thyroid cancer was initially inconsistent in men (14, 19). A pooled analysis of 22 prospective cohort studies from the US, Europe, and Asia reported an association of greater height, excess weight early in adulthood, and higher incidence of most major types of thyroid cancer, with the association of baseline BMI and thyroid cancer being stronger in men (20). A recent study in the US estimated that by 2015 one of every six PTCs diagnosed and two to three large PTCs diagnosed, among US adults over 60 years, were attributed to excessive weight (21). Likewise, in a recent meta-analysis that evaluated the impact of obesity and weight change in thyroid cancer risk, obesity was associated with a higher risk of thyroid cancer in women. Maintaining a healthy weight in both men and women was also associated with a reduced thyroid cancer risk (22).

This systematic review and meta-analysis aimed to examine the association between BMI and aggressive clinicopathologic features such as tumour-node-metastasis (TNM) stage, extrathyroidal extension (ETE), lymph node (LN) metastasis, and multifocality in patients with PTC. To our knowledge, there are no previous systematic reviews or meta-analyses that evaluated the association of BMI with aggressive clinicopathologic features in patients with PTC. Evidence of such associations would be extremely important in daily clinical practice, as weight loss programs could become part of PTC patient management.

## Methods

### Literature Search and Study Selection

We followed the Preferred Reporting Items for Systematic Reviews and Meta-Analyses guidelines (PRISMA) (23). The PRISMA 2009 checklist is shown in **Supplementary Table 1**. Two researchers (AE & KG) independently searched PubMed, EBSCO, and Cochrane library databases to identify potentially eligible articles that examine the association of BMI and TNM stage, ETE, LN, and multifocality in patients with PTC. Discrepancies were resolved by mediation and discussion with a third author (PE). The literature searches were conducted from inception to December 2020 and were restricted to English language publications; no date restriction was applied. Additional information concerning the search strategy is presented in **Supplementary Table 2**. First, each article’s title and abstracts identified through the search were examined, and then the full texts of potentially eligible articles were reviewed for evaluation. A reference list of relevant studies was screened to identify additional studies.

### Eligibility Criteria

We used the Participants, Interventions, Comparisons, Outcomes, and Study design (PICOS) approach (24) to identify included studies. Participants were only patients with PTC regardless of gender who underwent thyroid surgery. For the intervention, we considered overweight (BMI 25.0-29.9 kg/m² - WHO classification categories) and obese (BMI≥30 kg/m² - WHO-recommended BMI categories) who underwent total thyroidectomy. For the comparison, we considered studies with a control/comparison group of patients with normal weight (BMI<25.0 kg/m²/WHO-recommended BMI categories) who underwent total thyroidectomy. The chosen outcome was at least one of the following outcome measures: TNM stage, tumour size, LN metastasis, ETE, multifocality. For study design, any design was eligible for inclusion. Only human studies that investigated the association of BMI (weight in kg/height in meters squared) according to WHO classification and clinicopathologic features of PTC in patients who underwent total thyroidectomy, written in English, were considered for inclusion with no restriction regarding the time of publication. We excluded studies that grouped PTC with other histopathologic types of thyroid cancer, studies that self-reported height and weight, and studies that did not use the standard WHO BMI classification. Narrative review articles, dissertations, or theses, published abstracts, book chapters, points of view/expert opinions, animal studies, and case reports were not eligible for inclusion.

### Data Extraction and Quality Assessment

Two authors (AE & KG) extracted the data independently, and any discrepancy was resolved after consultation with a third author (PP). The extracted data collected included: first author, year of publication, country of origin, design of the study, sample size, characteristics of the study population (age, sex) and reported odds ratios (OR) (both adjusted and unadjusted) or rates of tumours size, ETE, multifocality, LN metastasis, and TNM stage. The methodological quality was performed using the Newcastle- Ottawa Scale (NOS) (25). The NOS is a validated scale to assess non-randomized control trials, and each study can be awarded up to nine stars. Each study is assessed on eight items, in three groups: quality of selection, comparability between the groups, and outcome. Studies with NOS values greater than six were considered moderate to high-quality studies (25).

### Statistical Analysis

The reported effect size estimates on the associations between BMI and TNM stage, tumour size, ETE, LN metastasis, multifocality in patients with PTC between the exposed group with the high BMI and the non-exposed group were pooled using meta-analysis. Reported effect size estimates expressed as odds ratio (OR) and its 95% confidence interval (CI) were used as the measuring standard to evaluate the strength of the association of PTC with the aggressive clinicopathologic features using fixed-effect and random-effect models. We measured heterogeneity among individual effect estimates, and we reported the P-value of the χ^2^-based Cochran Q test. The variation in estimates attributable to heterogeneity was quantified by the measure I^2^ metric for inconsistency (26) and I^2^ index (>50% indicating significant heterogeneity). If I^2^ > 50%, a random effects model was used to pool the results; otherwise, a fixed-effect model was applied (27). To further explore heterogeneity sources and sources of bias, original studies were stratified and pooled separately by BMI classification (overweight and obesity) and corresponding forest plots were constructed. We have also stratified studies that reported adjusted and unadjusted ORs separately, in order to assess the impact of confounding (28). The publication bias was examined by visual inspection of funnel plots and evaluated formally with Egger’s regression asymmetry test (29, 30). All statistical analyses were performed by STATA 14.0 software (STATA Corp, College Station, TX), and a two-tailed P value <0.05 was deemed statistically significant.

## Results

### Description of Studies

**Figure 1:**
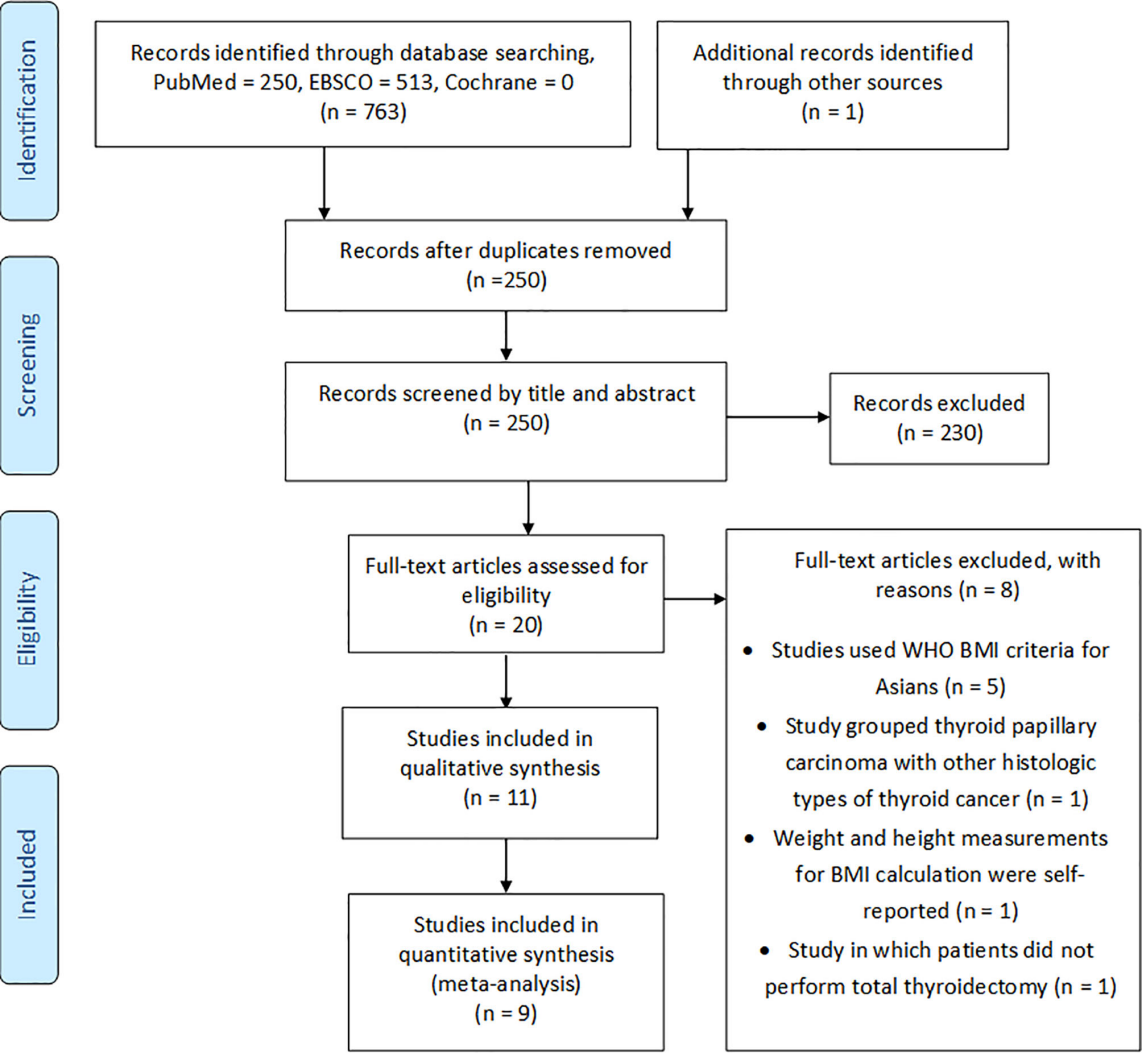
Study selection flow diagram.

From the initial electronic search, we identified 763 articles, while from a manual search of the bibliographies and reference lists of these articles, we identified one additional article. After duplication removal, there were 250 records to screen for titles and abstracts. Following titles and abstract screening, 230 articles were excluded based on the inclusion and exclusion criteria. The full texts of the remaining 20 articles were checked for eligibility, and of these, eight were excluded (31–38) for various reasons, as outlined in **Figure 1**. The 11 remaining articles (39–49) met the eligibility criteria and were included in this systematic review, where nine were enrolled in the quantitative meta-analysis (**Figure 1**).

### Characteristics of Studies Included

The characteristics of studies included in the qualitative synthesis and meta-analysis are described in **Tables 1** and **2**, respectively. All 11 studies included in this systematic review were retrospective cohort studies involving 26,196 participants. These studies were published from 2012 to 2020. There was significant variability in the number of patients included in each study ranging from 53 to 13,995. Most participants in the studies were females. From the studies included, four were from South Korea, four from China, and the remaining four from the US, France, Japan, and Pakistan. Most of the studies examined the association between BMI on clinicopathologic features, except from two studies (39, 45) that examined mostly post-operative outcomes according to BMI indices. In addition, two studies (41, 43) examined clinicopathologic features in PTMC.

**Table 1:** Characteristics of studies included in the qualitative synthesis.

First author	Country	Study design	Mean age (SD)	Female (%)	Total sample size	Total no of cases with aggressive features, n (%)	BMI categories n (%)			Main Findings
							Normal weight	Overweight	Obese	
**Advanced tumor-node-metastasis stage**										
Choi JS, 2014	South Korea	RC	n/a	87.4%	612^a^	201 (32.8%)	426 (69.6%)	141 (23.0%)	13 (2.1%)	A higher proportion of advanced TNM stages was found in the obese and overweight patients’ groups than the underweight and normal-weight groups.
Tresallet C, 2014	France	RC	49.9 ( ± 4.2)	78.5%	1216	252 (20.7%)	684 (56.3%)	356 (29.3%)	176 (14.5%)	Advanced TNM stage, stage 3, and stage 4 were more frequent with increasing BMI (18% in the normal-weight group, 22% in the overweight group, and 27% in the obese group (p<0.018).
**Tumour size**										
Tresallet C, 2014	France	RC	49.9 ( ± 4.2)	78.5%	1216	536 (44.1%)	684 (56.3%)	356 (29.3%)	176 (14.5%)	Tumour size was equivalent among the three evaluated BMI groups.
Kim SK, 2016	South Korea	RC	n/a	78.2%	5081	2518 (49.6%)	3255 (64.1%)	1582 (31.1%)	244 (4.8%)	Higher BMI associated with larger tumours, women p<0.001, men p<0.002.
Harari A, 2012	USA	RC	48.2	72.9%	443	n/a	175 (39.5%)	141 (31.8%)	99 (28.7%)	Obese patients reported larger tumors.
**Extrathyroidal extension**										
Choi JS, 2014	South Korea	RC	n/a	87.4%	612^a^	299 (48.9%)	426 (69.6%)	141 (23.0%)	13 (2.1%)	In PTMC patients, BMI was associated with ETE.
Kim SH, 2015	Japan	RC	47 ( ± 11.7)	84%	716	133 (19.6%)	481 (67.2%)	202 (28.2%)	33 (4.6%)	ETE was 16,8% in the group of patients with BMI < 24.9, 22.8% in the group of patients with BMI 25-29.9, and 18.2% in patients with BMI ≥ 30 (p=0.19).
Tresallet C, 2014	France	RC	49.9 ( ± 4.2)	78.5%	1216	299 (24.6%)	684 (56.3%)	356 (29.3%)	176 (14.5%)	Obese patients had a greater rate of microscopic ETE in patients with BMI < 30 (32% vs. 23%, p<0.016).
**Multifocality**										
Choi JS, 2014	South Korea	RC	n/a	87.4%	612^a^	189 (30.9%)	426 (69.6%)	141 (23.0%)	13 (2.1%)	Multifocality were predictive factors of advanced stage regardless of BMI in PTMC.
Tresallet C, 2014	France	RC	49.9 ( ± 4.2)	78.5%	1216	488 (40.1%)	684 (56.3%)	356 (29.3%)	176 (14.5%)	Multifocality rate was equivalent between the three BMI groups.
**Lymph node metastasis**										
Choi JS, 2014	South Korea	RC	n/a	87.4%	612^a^	188 (30.7%)	426 (69.6%)	141 (23.0%)	13 (2.1%)	The rates of metastatic LN did not differ among BMI groups in PTMC patients.
Zaman SU, 2018	Pakistan	RC	44.6 ( ± 14.3)	71.7%	53	24 (45.3%)	28 (52.8%)	n/a	25 (47.1%)	Higher frequencies of LN stage tumours in the obese group were reported but were not statistically significant.
Kim SH, 2015	Japan	RC	47 ( ± 11.7)	84%	716	342 (47.8%)	481 (67.2%)	202 (28.2%)	33 (4.6%)	Higher BMI was associated with more lymph node involvement (p<0.004) in patients < 45 years of age.
Tresallet C, 2014	France	RC	49.9 ( ± 4.2)	78.5%	1216	244 (20.1%)	684 (56.3%)	356 (29.3%)	176 (14.5%)	BMI was a risk factor for neck LN metastases (lateral or central compartment) and LN extracapsular spread when adjusted for confounding factors.

RC, Retrospective Cohort Study; SD, Standard Deviation; PTC, Papillary Thyroid Carcinoma; PTMC, Papillary Thyroid Micro carcinoma; n/a, not assessed; BMI, Body Mass Index; ETE, Extrathyroidal extension; LN, Lymph node; TNM; tumor-node-metastasis.

a Patients with PTMC.

**Table 2:** Characteristics of studies included in the meta-analysis.

First author	Country	Study design	Mean age (SD)	Female (%)	Total sample size	Total no of cases with aggressive features, n (%)	BMI categories n (%)		
							Normal weight	Overweight	Obese
**Advanced tumor-node-metastasis stage**									
Kim HJ, 2013	South Korea	RC	46 ( ± 13)	87%	2057	904 (43.9%)	1243 (60.4%)	661 (32.1%)	95 (4.6%)
Wu C, 2017	China	RC	46 ( ± 11.6)	76.8%	796	255 (32%)	403 (50.6%)	311 (39.1%)	64 (8.0%)
Feng JW, 2019	China	RC	45 ( ± 12)	74.6%	417	85 (20.4%)	247 (59.2%)	132 (31.7%)	25 (6.0%)
Liu Z, 2015	China	RC	n/a	84.4%^a^ 80.7%^b^	501^a^ 810^b^	237 (29.3%)	351^a^ (70%) 568^b^ (70.1%)	115^a^ (23%) 179^b^ (22.1%)	8^a^ (1.6%) 21^b^ (2.6%)
Harari A, 2012	USA	RC	48.2	72.9%	443	89 (20.0%)	175 (39.5%)	141 (31.8%)	127 (28.7%)
**Tumor Size**									
Kim HJ, 2013	South Korea	RC	46 ( ± 13)	87%	2057	1202 (58.4%)	1243 (60.4%)	661 (32.1%)	95 (4.6%)
Wu C, 2017	China	RC	46 ( ± 11.6)	76.8%	796	424 (53.3%)	403 (50.6%)	311 (39.1%)	64 (8.0%)
Feng JW, 2019	China	RC	45 ( ± 12)	74.6%	417	221(53.0%)	247 (59.2%)	132 (31.7%)	25 (6.0%)
Liu Z, 2015	China	RC	n/a	84.4%^a^ 80.7%^b^	810	309 (38.1%)	568 (70.1%)	179 (22.1%)	21 (2.6%)
Li CL, 2020	China	RC	42.9 ( ± 9.5)	78.7%	13995	3173 (22.7%)	8133 (58.1%)	4572 (32.7%)	846 (6.0%)
**Extrathyroidal extension**									
Kim HJ, 2013	South Korea	RC	46 ( ± 13)	87%	2057	1592 (77.4%)	1243 (60.4%)	661 (32.1%)	90 (4.6%)
Wu C, 2017	China	RC	46 ( ± 11.6)	76.8%	796	308 (38.7%)	403 (50.6%)	311 (39.1%)	64 (8.0%)
Feng J, 2019	China	RC	45 ( ± 12)	74.6%	417	63 (15.1%)	247 (59.2%)	132 (31.7%)	25 (6.0%)
Liu Z, 2015	China	RC	n/a	84.4%^a^ 80.7%^b^	501^a^ 810^b^	327 (40.4%)	351^a^ (70%) 568^b^ (70.1%)	115^a^ (23%) 179^b^ (22.1%)	8^a^ (1.6%) 21^b^ (2.6%)
Li CL, 2020	China	RC	42.9 ( ± 9.5)	78.7%	13995	3735 (26.7%)	8133 (58.1%)	4572 (32.7%)	846 (6.0%)
Zaman SU, 2018	Pakistan	RC	44.6 ( ± 14.3)	71.7%	53	12 (22.6%)	28 (52.8%)	n/a	25 (47.1%)
Kim SK, 2016	South Korea	RC	n/a	78.2%	5081	3440 (67.7%)	3255 (64.1%)	1582 (31.1%)	244 (4.8%)
**Multifocality**									
Kim HJ, 2013	South Korea	RC	46 ( ± 13)	87%	2057	640 (31.1%)	1243 (60.4%)	661 (32.1%)	90 (4.6%)
Wu C, 2017	China	RC	46 ( ± 11.6)	76.8%	796	348 (43.7%)	403 (50.6%)	311 (39.1%)	64 (8.0%)
Feng JW, 2019	China	RC	45 ( ± 12)	74.6%	417	123 (29.5%)	247 (59.2%)	132 (31.7%)	25 (6.0%)
Liu Z, 2015	China	RC	n/a	84.4%^a^ 80.7%^b^	501^a^ 810^b^	316 (39.0%)	351^a^ (70%) 568^b^ (70.1%)	115^a^ (23%) 179^b^ (22.1%)	8^a^ (1.6%) 21^b^ (2.6%)
Li CL, 2020	China	RC	42.9 ( ± 9.5)	78.7%	13995	5647 (40.4%)	8133 (58.1%)	4572 (32.7%)	846 (6.0%)
Zaman SU, 2018	Pakistan	RC	44.6 ( ± 14.3)	71.7%	53	13 (24.5%)	28 (52.8%)	n/a	25 (47.1%)
Kim SK, 2016	South Korea	RC	n/a	78.2%	5081	1515 (29.8%)	3255 (64.1%)	1582 (31.1%)	244 (4.8%)
Kim SH, 2015	Japan	RC	47 ( ± 11.7)	84%	716	211 (29.5%)	481 (67.2%)	202 (28.2%)	33 (4.6%)
**Lymph node metastasis**									
Kim HJ, 2013	South Korea	RC	46 ( ± 13)	87%	2057	774 (37.6%)	1243 (60.4%)	661 (32.1%)	95 (4.6%)
Wu C, 2017	China	RC	46 ( ± 11.6)	76.8%	796	689 (86.6%)	403 (50.6%)	311 (39.1%)	64 (8.0%)
Feng JW, 2019	China	RC	45 ( ± 12)	74.6%	417	203 (48.7%)	247 (59.2%)	132 (31.7%)	25 (6.0%)
Liu Z, 2015	China	RC	N/A	84.4%^a^ 80.7%^b^	501^a^ 810^b^	347 (42.8%)	351^a^ (70%) 568^b^ (70.1%)	115^a^ (23%) 179^b^ (22.1%)	8^a^ (1.6%) 21^b^ (2.6%)
Li CL, 2020	China	RC	42.9 ( ± 9.5)	78.7%	13995	5869 (41.9%)	8133 (58.1%)	4572 (32.7%)	846 (6.0%)
Kim SK, 2016	South Korea	RC	n/a	78.2%	5081	3183 (62.6%)	3255 (64.1%)	1582 (31.1%)	244 (4.8%)

RC, Retrospective Cohort Study; SD, Standard Deviation; PTC, Papillary Thyroid Carcinoma; PTMC, Papillary Thyroid Micro carcinoma; n/a, not assessed; BMI, Body Mass Index/kg/m².

a Patients with PTMC.

b Patients with PTC.

### Quality Assessment and Publication Bias

The risk of bias for the studies included was evaluated using the NOS tool. The quality scores of the 11 included studies are shown in **Table 3**. The maximum quality score is 9 and the range of scores was from 4 to 7. The publication bias of the studies was determined by visual inspection of funnel plots for asymmetry or outliers and then evaluated formally with Egger’s regression asymmetry test. The funnel plots showed reasonable symmetry, with no evidence of publication bias, except for meta-analyses on the association between overweight and ETE as well as obesity and ETE where shapes of the funnel plots appeared asymmetrical (**Supplementary File 1**). Likewise, no evidence of publication bias was observed in any meta-analyses (all P > 0.05), except for studies on ETE for both overweight and obesity patients (P_Egger’s test <_0.05) (**Supplementary Table 3**).

**Table 3:** Quality assessment of cohort studies.

Author, year	Selection				Comparability	Outcome			Total quality score
	(1) Representativeness of the exposed cohort	(2) Selection of the non-exposed cohort	(3) Ascertainment of exposure	(4) Demonstration that outcome of interest was not present at start of study	(1) Comparability of cohorts on the basis of the design or analysis^a^	(1) Assessment of outcome	(2) Was follow-up long enough for outcomes to occur?	(3) Adequacy of follow up of cohorts	
Kim HJ, 2013	★	★	★		★★	★	★		7
Zaman SU, 2018		★	★			★	★		4
Kim SH, 2015	★	★	★		★	★	★		6
Tresallet C, 2014	★	★	★			★	★		5
Kim SK, 2016	★	★	★		★★	★	★		7
Wu C, 2017	★	★	★		★★	★	★		7
Feng JW, 2019	★	★	★		★★	★	★		7
Harari A, 2012	★	★	★		★★	★	★		7
Liu Z, 2015	★	★	★		★★	★	★		7
Choi JS, 2014	★	★	★		★★	★	★		7
Li CL, 2020	★	★	★		★★	★	★		7

a A maximum of 2 stars can be awarded for this item. A study controlling for age receives one star, and a study controlling for other major risk factors receives an additional star.

### BMI and Advanced TNM Stage

**Figure 2:**
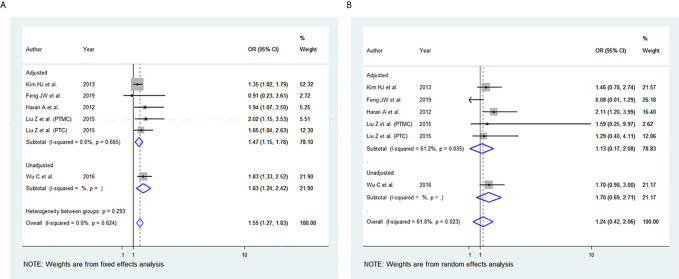
Forest plots for the meta-analysis for the association between Body Mass Index and Advanced Tumor-Node-Metastasis (TNM) stage. **(A)** Meta-analysis between overweight and advanced TNM stage. **(B)** Meta-analysis between obesity and advanced TNM stage.

Among the 11 studies included, six studies were eligible for meta-analysis for the association between BMI and advanced TNM stage. A fixed-effect model was applied to analyse the data, and the overall OR for overweight patients was 1.55 (95% CI: 1.27-1.83), and there was no heterogeneity among study results (I^2^= 0.0%, p=0.617) (**Figure 2A**). For the obese group of patients, the overall OR was 1.24 (95% CI: 0.42-2.06), and there was statistically significant heterogeneity among study results (I^2^ = 61.8%, p=0.023) (**Figure 2B**).

### BMI and Tumour Size

**Figure 3:**
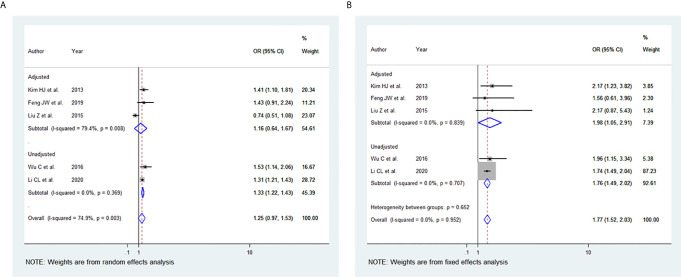
Forest plots for the meta-analysis for the association between Body Mass Index and Tumour -Size. **(A)** Meta-analysis between overweight and tumour size. **(B)** Meta-analysis between obesity and tumour size.

Among the 11 studies included, five studies were eligible for meta-analysis for the association between BMI and tumour size. A random-effect model was applied to analyse the data, and the overall OR for the overweight patients was 1.25 (95% CI: 0.97-1.53), and there was statistically significant high heterogeneity among study results (I^2^ = 74.9%, p=0.003) (**Figure 3A**). For the obese group of patients, the overall OR was 1.77 (95% CI: 1.52-2.03) using the fixed-effect model, and there was no heterogeneity among study results (I^2^ = 0.0%, p=0.952) **(** **Figures 3B** **).** The results of the studies that were not included in our meta-analysis are shown in **Table 2**.

### BMI and Extrathyroidal Extension (ETE)

**Figure 4:**
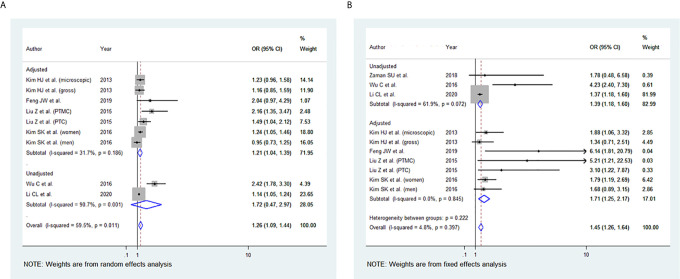
Forest plots for the meta-analysis for the association between Body Mass Index and Extrathyroidal Extension (ETE). **(A)** Meta-analysis between overweight and ETE. **(B)** Meta-analysis between obesity and ETE.

Among the 11 studies included, seven studies were eligible for meta-analysis for the association between BMI and ETE. A random-effect model was applied to analyse the data and the overall OR for overweight patients was 1.26 (95% CI: 1.09-1.44), and there was statistically significant heterogeneity among study results (I^2^ = 59.5%, p=0.011) (**Figure 4A**). For the obese group of patients, the overall OR was 1.45 (95% CI: 1.26-1.64) using the fixed-effect model, and there was no significant statistical heterogeneity among study results (I^2^ = 4.8%, p=0.397) **(** **Figures 4B** **)**. The results of the studies that were not included in our meta-analysis are described in **Table 2**.

### BMI and Multifocality

**Figure 5:**
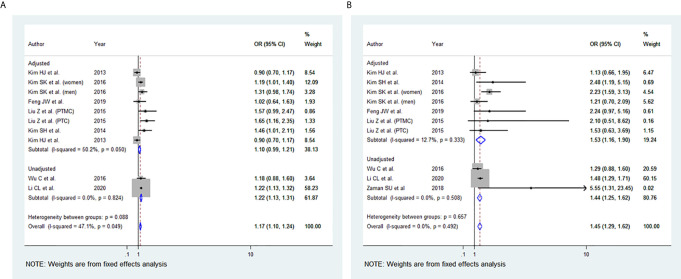
Forest plots for the meta-analysis for the association between Body Mass Index and Multifocality. **(A)** Meta-analysis between overweight and multifocality. **(B)** Meta-analysis between obesity and multifocality.

Among the 11 studies included, eight studies were eligible for meta-analysis for the association between BMI and multifocality. A fixed-effect model was applied to analyse the data, and the overall OR for overweight patients was 1.17 (95% CI: 1.10-1.24), and there was marginal statistical heterogeneity among study results (I^2 ^= 47.1%, p=0.049) (**Figure 5A**). For the obese group of patients, the overall OR was 1.45 (95% CI: 1.29-1.62), and there was no heterogeneity among study results (I^2 ^= 0.0%, p=0.492) **(** **Figures 5B** **).** The results of the studies that were not included in our meta-analysis are presented in **Table 2**.

### BMI and Lymph Node (LN) Metastasis

**Figure 6:**
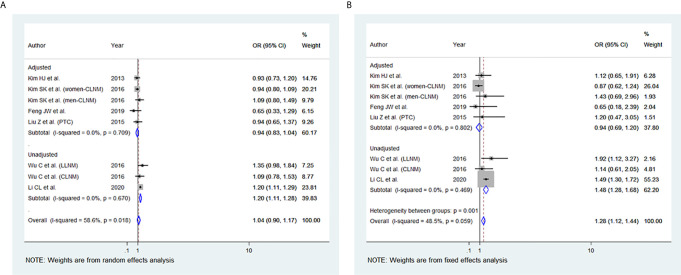
Forest plots for the meta-analysis for the association between Body Mass Index and lymph node (LN) Metastasis. **(A)** Meta-analysis between overweight and LN Metastasis. **(B)** Meta-analysis between obesity and LN Metastasis.

Among the 11 studies included, six studies were eligible for meta-analysis for the association between BMI and LN Metastasis. A random-effect model was applied to analyse the data, and the overall OR for overweight patients was 1.04 (95% CI: 0.90-1.17), and there was statistically significant heterogeneity among study results (I^2 ^= 58.6%, p=0.018) (**Figure 6A**). For the obese group of patients, the overall OR was 1.28 (95% CI: 1.12-1.44) under the fixed-effect model, and there was no statistical heterogeneity among study results (I^2 ^= 48.5%, p=0.059) **(** **Figures 6B** **).** The results of the studies that were not included in our meta-analysis are shown in **Table 2**.

## Discussion

The present study is the first systematic review and meta-analysis comprehensively evaluating the association of BMI with aggressive clinicopathologic features in patients with PTC. Eleven retrospective cohort studies involving 26,196 participants were included. Our findings showed that elevated BMI was significantly associated with ETE in both overweight (OR 1.26, 95% CI: 1.09-1.44) and obesity group (OR 1.45, 95% CI: 1.26-1.64) as well as with multifocality in both overweight (OR 1.17, 95% CI: 1.10-1.24) and obese patients (OR 1.45, 95% CI: 1.29-1.62). Likewise, obesity was significantly associated with increased tumour size (OR 1.77, 95% CI: 1.52-2.03) and with LN metastasis (OR 1.28, 95% CI: 1.12-1.44), whereas being overweight was significantly associated with a more advanced TNM stage (OR 1.55, 95% CI: 1.27-1.83).

Thyroid cancer incidence has been rising, along with obesity rates over the past decade (50). Thyroid cancer, the most common endocrine cancer, has become the fastest-growing cancer globally (5). The rise in the incidence of obesity has preceded the increase in thyroid cancer rate by a few years. It is noted that after a certain point, the rise of the two incidences became parallel (10). The majority of thyroid cancers are PTC, accounting for more than 75-85% of thyroid cancer cases and are considered slow-growing well-differentiated cancers with an overall 96.6% survival rate (51). However, in recent years there was an increase in more aggressive tumours, and it was recently shown that even small multifocal papillary thyroid carcinoma has an increased incidence of aggressive features (6–8, 52).

The possible underlining mechanisms that could explain the association between aggressive features of thyroid cancer and obesity, have been widely investigated. Obesity leads to insulin resistance (IR), which has been linked to thyroid cancer through insulin-like growth factor-1 (IGF-1) and thyroid-stimulating hormone (TSH) signalling, inflammation, or oxidative stress (9). Hyperinsulinemia leads to decreased synthesis of IGF- binding proteins (IGFBPs), resulting in an increase of free circulating IGF-1. Insulin and IGF-1 have mitogenic effects and have also been associated with various other types of cancer (9). The increase of IGF-1 levels *in vitro* results in increased cell proliferation, and in thyroid cancer cells, IGF receptors were found to be overexpressed compared to normal thyroid cells (53).

Adiponectin, a collagen-like polypeptide, is a hormone involved in various metabolic processes, including insulin-sensitizing and anti-inflammatory processes. It is secreted from adipose tissue and acts through two transmembrane receptors (Adipo R1 and Adipo R2). It also acts as an anti-tumour-agent by inhibiting cell proliferation and angiogenesis and increasing apoptosis. In many studies, adiponectin was found to be inversely related to BMI. Although, a previous study revealed lower circulating adiponectin levels in any form of thyroid cancer (54), yet, a large multicentre prospective study confirmed these findings only in women (55).

Another possible mechanism that has been proposed to explain the association between obesity and thyroid cancer is the elevated TSH that has been associated with both obesity and advanced tumour stage (10). TSH levels were assessed in most studies included, and some reported that patients with abnormal results were excluded. However, three studies did not provide information or account for elevated TSH as a confounding factor (39, 41, 47). Hypothyroidism with elevated TSH can cause excess water accumulation, and as this interferes with BMI measurements, thus it is important to exclude patients with hypothyroidism (56). Although obesity leads to elevated fasting blood glucose or diabetes mellitus and elevated cholesterol levels, there is no clear association between these metabolic parameters and thyroid cancer (9). Additional studies are needed to explain this association. It is noted that few of the included studies measured these metabolic factors (40, 42, 44–46), however, most of them did not.

Although the exact molecular mechanisms of the association among increased BMI and larger tumours or tumours with the more advanced stage are still unclear, recent data support the role of adipokines such as leptin and adiponectin. Leptin plays a key role in homeostasis and energy balance, and it is elevated when there is an increase in fat mass. Leptin expression was correlated in patients with PTC with larger tumours, nodal metastasis, and advanced stage (57). Overexpression of leptin and its receptors in PTC patients was associated with aggressive phenotypes in tumour size and LN metastasis (58). Also, an important role of leptin in tumour pathogenesis in PTC is mediated *via* the PI_3_K/AKT pathway through the membrane receptor Ob-R (59).

In our study, elevated BMI was significantly associated with the advanced TNM stage in most studies included. The same observation was seen in our meta-analysis of the advanced TNM stage in the overweight group and obesity group (**Figure 2**). However, two of the studies included (40, 44) revealed no evidence of association between TNM and elevated BMI. This may be attributed to the small number of obese patients included and thus likely being underpowered. In addition, high BMI has been associated with tumour size in the overweight and obesity group, yet the results were statistically significant only in the obesity group (**Figure 3**). The lack of association may be attributed to the marked variability and non-uniformity in tumour size reporting in studies included. In our meta-analysis, LN metastasis was associated with higher BMI in the overweight and obesity group, but the results were statistically significant only for the obesity group. The lack of association may be attributed to the possible preoperative diagnostic difficulty in these patients leading to inadequate surgery, subsequently leading to false-negative outcomes.

In our meta-analysis, the ETE was associated with higher BMI in the overweight and the obesity group (**Figure 4**). Microscopic ETE and gross ETE were assessed by two studies (42, 45). Kim HJ et al. reported a strong association with microscopic ETE in obese patients (42), while Tressallet C et al. reported that obese patients had a greater rate of microscopic ETE (45). The association of obesity and ETE was also strong in the two studies that included Papillary Thyroid Micro carcinoma (PTMC) patients (41, 43). Multifocality was associated with higher BMI in the overweight and obesity group (**Figure 5**). However, in the study by Kim SK et al., a positive association between multifocality with higher BMI and Body Surface Area (BSA) was shown only in women and not in men, whereas higher BMI and BSA were independent predictors for multifocality (49). A positive association was also seen in PTMC patients where multifocality and tumour size were predictors of advanced-stage regardless of BMI (43).

Previous studies indicate a stronger association of BMI and thyroid cancer in women than men, with the risk estimation to be from 1.1 to 2.3 in men and 1.0 to 7.4 in women (60). However, this may not be a real effect, but since thyroid carcinoma is less common in men, many studies have lower statistical power (10). Thyroid cancer and obesity rates are higher in women (15) and thyroid cancer is three to four times as common in women, with increasing incidences during the reproductive years (9, 10, 12, 61). The synthesis of oestrogens from peripheral aromatization in the adipose tissue may be implicated in the underlying process, and an exceeded threshold of estrogenic load, with a possible altered immune response and augmented mitogenic processes, may be responsible for the increase in thyroid cancer rates in women (9, 10). In addition, higher circulating oestradiol, the strongest produced oestrogen, has been reported in obese postmenopausal women compared to normal-weight women (62), and this can cause cell proliferation in PTC cells *in vitro* (63). A potent mitogenic effect of oestrogen in thyroid cancer has been also reported (64).

There are several limitations in our study that must be considered when interpreting our results. To enhance comparability between studies, we excluded studies that applied different BMI classification categories (e.g., WHO Asian BMI cut points) other than the WHO-recommended BMI categories for relative weight classification as well as studies that grouped PTC with other histologic types of thyroid cancer, which could limit our findings. Of note, previous studies that examined the association between BMI and aggressiveness of differentiated thyroid cancer (DTC) found no significant associations between BMI with any aggressive clinicopathologic features of thyroid cancer (31, 65, 66). However, the inclusion of such studies would increase the heterogeneity between studies included. Furthermore, the original studies’ retrospective design permits for a considerable degree of selection bias that might affect our conclusions. Also, uncontrolled confounding factors such as age, sex, duration of obesity, weight history, exercise, smoking, alcohol intake, genetic and environmental differences between the study populations could lead to exaggerating or underestimating the association. Likewise, other confounding factors implicated in the underlying mechanisms, including hormones and biological factors such as insulin, leptin, adiponectin, IGF-1, and other cytokines were not assessed, which might affect the observed association. In addition, information on the patient’s medical history, such as diabetes, hypercholesterolemia, cardiovascular disease, or hypothyroidism, was not always available. Also, the number of patients in some studies was small, and probably those studies were underpowered to detect any significant differences. Most of the studies collected data from a single medical centre, so the study population was not always representative of the whole population. It is important to note that few of the studies included were of low to moderate methodological quality, which may jeopardise the validity of their results. Although BMI is the most mentioned method of assessing obesity, waist to hip ratio, skinfold thickness, and intra-abdominal fat are also good indexes for assessing obesity. Only one study provided BSA measurements as an assessment of adiposity (49). Lastly, follow-up was not universal for all studies, although only a few studies did not report a follow-up period.

## Conclusion

To the best of our knowledge, this is the first systematic review and meta-analysis to identify whether adiposity is related to aggressive clinicopathologic features in patients with PTC. Higher BMI is found to be associated with ETE and multifocality. Obesity was associated with tumor size and LN metastasis, whereas being overweight was associated with the advanced TNM stage. More studies with a larger number of participants are needed to examine and further clarify the association of increased BMI, advanced TNM stage, and LN metastasis. Given the current obesity epidemic and the solid evidence that adipose tissue supports both tumorigenesis and metastasis (67, 68), a successful public health program promoting a healthy lifestyle and weight loss may help with not only decreasing the incidence of thyroid cancer but also combat its aggressiveness.

## Data Availability Statement

The original contributions presented in the study are included in the article/**Supplementary Material**. Further inquiries can be directed to the corresponding author.

## Author Contributions

AE carried out literature searches, appraised the articles, summarized the results, prepared the tables, wrote the manuscript and interpreted the results. KG supervised the process, carried out literature searches, appraised the articles, performed the meta-analysis, prepared the tables, wrote the manuscript and interpreted the results. IM interpreted the results. PE appraised the articles, summarized the results, prepared the tables, wrote the manuscript and interpreted the results. PP conceived the theme, supervised the process, wrote the manuscript and interpreted the results. All authors contributed to the article and approved the submitted version.

## Conflict of Interest

The authors declare that the research was conducted in the absence of any commercial or financial relationships that could be construed as a potential conflict of interest.

## References

[1] Ferlay J Steliarova-Foucher E Lortet-Tieulent J Rosso S Coebergh J-WW Comber H. Cancer Incidence and Mortality Patterns in Europe: Estimates for 40 Countries in 2012. Eur J cancer (2013) 49(6):1374–403. 10.1016/j.ejca.2012.12.027 23485231

[2] American_Cancer_Society. Key Statistics for Thyroid Cancer (2021). Available at: https://www.cancer.org/cancer/thyroid-cancer/about/key-statistics.html.

[3] Davies L Welch HG. Increasing Incidence of Thyroid Cancer in the United States, 1973-2002. Jama (2006) 295(18):2164–7. 10.1001/jama.295.18.2164 16684987

[4] Lim H Devesa SS Sosa JA Check D Kitahara CM. Trends in Thyroid Cancer Incidence and Mortality in the United States, 1974-2013. Jama (2017) 317(13):1338–48. 10.1001/jama.2017.2719 PMC8216772 28362912

[5] Siegel RL Miller KD Jemal A. Cancer Statistics, 2016. CA: Cancer J Clin (2016) 66(1):7–30. 10.3322/caac.21332 26742998

[6] Enewold L Zhu K Ron E Marrogi AJ Stojadinovic A Peoples GE. Rising Thyroid Cancer Incidence in the United States by Demographic and Tumor Characteristics, 1980-2005. Cancer Epidemiol Prev Biomarkers (2009) 18(3):784–91. 10.1158/1055-9965.EPI-08-0960 PMC2676561 19240234

[7] Pazaitou-Panayiotou K Iliadou P Chrisoulidou A Mitsakis P Doumala E Fotareli A. The Increase in Thyroid Cancer Incidence is Not Only Due to Papillary Microcarcinomas: A 40-Year Study in 1 778 Patients. Exp Clin Endocrinol Diabetes (2013) 121(07):397–401. 10.1055/s-0033-1345125 23696480

[8] Segovia IG Gallowitsch H Kresnik E Kumnig G Igerc I Matschnig S. Descriptive Epidemiology of Thyroid Carcinoma in Carinthia, Austria: 1984–2001. Histopathologic Features and Tumor Classification of 734 Cases Under Elevated General Iodination of Table Salt Since 1990: Population-Based Age-Stratified Analysis on Thyroid Carcinoma Incidence. Thyroid (2004) 14(4):277–86. 10.1089/105072504323030933 15142361

[9] Pazaitou-Panayiotou K Polyzos S Mantzoros C. Obesity and Thyroid Cancer: Epidemiologic Associations and Underlying Mechanisms. Obes Rev (2013) 14(12):1006–22. 10.1111/obr.12070 24034423

[10] Pappa T Alevizaki M. Obesity and Thyroid Cancer: A Clinical Update. Thyroid (2014) 24(2):190–9. 10.1089/thy.2013.0232 23879222

[11] Tremmel M Gerdtham U-G Nilsson PM Saha S. Economic Burden of Obesity: A Systematic Literature Review. Int J Environ Res Public Health (2017) 14(4):435. 10.3390/ijerph14040435 PMC5409636 28422077

[12] NCD_Risk_Factor_Collaboration. Trends in Adult Body-Mass Index in 200 Countries From 1975 to 2014: A Pooled Analysis of 1698 Population-Based Measurement Studies With 19· 2 Million Participants. Lancet (2016) 387(10026):1377–96. 10.1016/S0140-6736(16)30054-X PMC7615134 27115820

[13] Renehan AG Tyson M Egger M Heller RF Zwahlen M. Body-Mass Index and Incidence of Cancer: A Systematic Review and Meta-Analysis of Prospective Observational Studies. Lancet (2008) 371(9612):569–78. 10.1016/S0140-6736(08)60269-X 18280327

[14] Kitahara CM Platz EA Freeman LEB Hsing AW Linet MS Park Y. Obesity and Thyroid Cancer Risk Among US Men and Women: A Pooled Analysis of Five Prospective Studies. Cancer Epidemiol Prev Biomarkers (2011) 20(3):464–72. 10.1158/1055-9965.EPI-10-1220 PMC3079276 21266520

[15] Polyzos SA Kita M Avramidis A. Thyroid Nodules-Stepwise Diagnosis and Management. HORMONES-ATHENS- (2007) 6(2):101. 10.14310/horm.2002.111107 17704042

[16] Davies L Ouellette M Hunter M Welch HG. The Increasing Incidence of Small Thyroid Cancers: Where Are the Cases Coming From? Laryngoscope (2010) 120(12):2446–51. 10.1002/lary.21076 21108428

[17] Tumminia A Vinciguerra F Parisi M Graziano M Sciacca L Baratta R. Adipose Tissue, Obesity and Adiponectin: Role in Endocrine Cancer Risk. Int J Mol Sci (2019) 20(12):2863. 10.3390/ijms20122863 PMC6628240 31212761

[18] Schmid D Ricci C Behrens G Leitzmann M. Adiposity and Risk of Thyroid Cancer: A Systematic Review and Meta-Analysis. Obes Rev (2015) 16(12):1042–54. 10.1111/obr.12321 26365757

[19] Engeland A Tretli S Akslen L Bjørge T. Body Size and Thyroid Cancer in Two Million Norwegian Men and Women. Br J cancer (2006) 95(3):366–70. 10.1038/sj.bjc.6603249 PMC2360634 16832414

[20] Kitahara CM McCullough ML Franceschi S Rinaldi S Wolk A Neta G. Anthropometric Factors and Thyroid Cancer Risk by Histological Subtype: Pooled Analysis of 22 Prospective Studies. Thyroid (2016) 26(2):306–18. 10.1089/thy.2015.0319 PMC4754509 26756356

[21] Kitahara CM Pfeiffer RM Sosa JA Shiels MS. Impact of Overweight and Obesity on US Papillary Thyroid Cancer Incidence Trends (1995–2015). JNCI: J Natl Cancer Inst (2020) 112(8):810–7. 10.1093/jnci/djz202 PMC7825478 31638139

[22] Youssef MR Reisner AS Attia AS Hussein MH Omar M LaRussa A. Obesity and the Prevention of Thyroid Cancer: Impact of Body Mass Index and Weight Change on Developing Thyroid Cancer–Pooled Results of 24 Million Cohorts. Oral Oncol (2021) 112:105085. 10.1016/j.oraloncology2020.105085 33171329

[23] Moher D Liberati A Tetzlaff J Altman DG Group P. Preferred Reporting Items for Systematic Reviews and Meta-Analyses: The PRISMA Statement. PloS Med (2009) 6(7):e1000097. 10.1371/journal.pmed.1000097 19621072 PMC2707599

[24] O’Connor D Green S Higgins JP. 5 Defining the Review Question and Developing Criteria for Including Studies. In Cochrane Handbook for Systematic Reviews of Interventions (Higgins J. P. Green S.). 10.1002/9780470712184.ch5

[25] Stang A. Critical Evaluation of the Newcastle-Ottawa Scale for the Assessment of the Quality of Nonrandomized Studies in Meta-Analyses. Eur J Epidemiol (2010) 25(9):603–5. 10.1007/s10654-010-9491-z 20652370

[26] Higgins JP Thompson SG. Quantifying Heterogeneity in a Meta-Analysis. Stat Med (2002) 21(11):1539–58. 10.1002/sim.1186 12111919

[27] Higgins JP Thompson SG Deeks JJ Altman DG. Measuring Inconsistency in Meta-Analyses. BMJ (Clinical Res ed). (2003) 327(7414):557–60. 10.1136/bmj.327.7414.557 PMC192859 12958120

[28] DerSimonian R Laird N. Meta-Analysis in Clinical Trials Revisited. Contemp Clin trials (2015) 45:139–45. 10.1016/j.cct.2015.09.002 PMC4639420 26343745

[29] Egger M Smith GD Schneider M Minder C. Bias in Meta-Analysis Detected by a Simple, Graphical Test. BMJ (Clinical Res ed). (1997) 315(7109):629–34. 10.1136/bmj.315.7109.629 PMC2127453 9310563

[30] Sterne JA Egger M Smith GD. Investigating and Dealing With Publication and Other Biases in Meta-Analysis. BMJ (Clinical Res ed). (2001) 323(7304):101–5. 10.1136/bmj.323.7304.101 PMC1120714 11451790

[31] Paes JE Hua K Nagy R Kloos RT Jarjoura D Ringel MD. The Relationship Between Body Mass Index and Thyroid Cancer Pathology Features and Outcomes: A Clinicopathological Cohort Study. J Clin Endocrinol Metab (2010) 95(9):4244–50. 10.1210/jc.2010-0440 PMC2936072 20519347

[32] Kim J-Y Jung E-J Jeong S-H Jeong C-Y Ju Y-T Lee Y-J. The Indices of Body Size and Aggressiveness of Papillary Thyroid Carcinoma. J Korean Surg Soc (2011) 80(4):241. 10.4174/jkss.2011.80.4.241 22066042 PMC3204682

[33] Dieringer P Klass EM Caine B Smith-Gagen J. Associations Between Body Mass and Papillary Thyroid Cancer Stage and Tumor Size: A Population-Based Study. J Cancer Res Clin Oncol (2015) 141(1):93–8. 10.1007/s00432-014-1792-2 PMC11823684 25113832

[34] Kwon H Kim M Choi YM Jang EK Jeon MJ Kim WG. Lack of Associations Between Body Mass Index and Clinical Outcomes in Patients With Papillary Thyroid Carcinoma. Endocrinol Metab (2015) 30(3):305. 10.3803/EnM.2015.30.3.305 PMC4595355 25433662

[35] Yu S-T Chen W Cai Q Liang F Xu D Han P. Pretreatment BMI is Associated With Aggressive Clinicopathological Features of Papillary Thyroid Carcinoma: A Multicenter Study. Int J Endocrinol (2017) 2017. 10.1155/2017/5841942 PMC5632484 29085428

[36] Zhao S Jia X Fan X Zhao L Pang P Wang Y. Association of Obesity With the Clinicopathological Features of Thyroid Cancer in a Large, Operative Population: A Retrospective Case-Control Study. Medicine (2019) 98(50). 10.1097/MD.0000000000018213 PMC6922396 31852078

[37] Wang H Wang P Wu Y Hou X Peng Z Yang W. Correlation Between Obesity and Clinicopathological Characteristics in Patients With Papillary Thyroid Cancer: A Study of 1579 Cases: A Retrospective Study. PeerJ (2020) 8:e9675. 10.7717/peerj.9675 33194342 PMC7485482

[38] Chung YS Lee J-H Lee YD. Is Body Mass Index Relevant to Prognosis of Papillary Thyroid Carcinoma? A Clinicopathological Cohort Study. Surg Today (2017) 47(4):506–12. 10.1007/s00595-016-1417-2 27654453

[39] Harari A Endo B Nishimoto S Ituarte PH Yeh MW. Risk of Advanced Papillary Thyroid Cancer in Obese Patients. Arch Surg (2012) 147(9):805–11. 10.1001/archsurg.2012.713 22914989

[40] Zaman SU Awan MS Sulaiman MA. Obesity and High Risk Pathological Features of Papillary Thyroid Carcinoma: A Retrospective Analysis of a University Hospital in Pakistan. Gulf J Oncol (2018) 5(27):6. 30145545

[41] Liu Z Maimaiti Y Yu P Xiong Y Zeng W Li X. Correlation Between Body Mass Index and Clinicopathological Features of Papillary Thyroid Microcarcinoma. Int J Clin Exp Med (2015) 8(9):16472. 26629173 PMC4659061

[42] Kim HJ Kim NK Choi JH Sohn SY Kim SW Jin SM. Associations Between Body Mass Index and Clinico-Pathological Characteristics of Papillary Thyroid Cancer. Clin endocrinol (2013) 78(1):134–40. 10.1111/j.1365-2265.2012.04506.x 22812676

[43] Choi JS Kim E-K Moon HJ Kwak JY. Higher Body Mass Index may be a Predictor of Extrathyroidal Extension in Patients With Papillary Thyroid Microcarcinoma. Endocrine (2015) 48(1):264–71. 10.1007/s12020-014-0293-z 24858734

[44] Feng J-W Yang X-H Wu B-Q Sun D-L Jiang Y Qu Z. Influence of Body Mass Index on the Clinicopathologic Features of Papillary Thyroid Carcinoma. Ann Otol Rhinol Laryngol (2019) 128(7):625–32. 10.1177/0003489419834314 30841713

[45] Trésallet C Seman M Tissier F Buffet C Lupinacci RM Vuarnesson H. The Incidence of Papillary Thyroid Carcinoma and Outcomes in Operative Patients According to Their Body Mass Indices. Surgery (2014) 156(5):1145–52. 10.1016/j.surg.2014.04.020 24878452

[46] Wu C Wang L Chen W Zou S Yang A. Associations Between Body Mass Index and Lymph Node Metastases of Patients With Papillary Thyroid Cancer: A Retrospective Study. Medicine (2017) 96(9). 10.1097/MD.0000000000006202 PMC5340448 28248875

[47] Kim S-H Park HS Kim K-H Yoo H Chae B-J Bae J-S. Correlation Between Obesity and Clinicopathological Factors in Patients With Papillary Thyroid Cancer. Surg Today (2015) 45(6):723–9. 10.1007/s00595-014-0984-3 25059345

[48] Li C Dionigi G Zhao Y Liang N Sun H. Influence of Body Mass Index on the Clinicopathological Features of 13,995 Papillary Thyroid Tumors. J endocrinol Invest (2020) 43(9):1283–99. 10.1007/s40618-020-01216-6 32166701

[49] Kim SK Woo J-W Park I Lee JH Choe J-H Kim J-H. Influence of Body Mass Index and Body Surface Area on the Behavior of Papillary Thyroid Carcinoma. Thyroid (2016) 26(5):657–66. 10.1089/thy.2015.0632 26959390

[50] Simard EP Ward EM Siegel R Jemal A. Cancers With Increasing Incidence Trends in the United States: 1999 Through 2008. CA: Cancer J Clin (2012) 62(2):118–28. 10.3322/caac.20141 22281605

[51] Haugen BR Alexander EK Bible KC Doherty GM Mandel SJ Nikiforov YE. American Thyroid Association Management Guidelines for Adult Patients With Thyroid Nodules and Differentiated Thyroid Cancer: The American Thyroid Association Guidelines Task Force on Thyroid Nodules and Differentiated Thyroid Cancer. Thyroid (2016) 26(1):1–133. 10.1089/thy.2015.0020 26462967 PMC4739132

[52] Papaioannou C Lamnisos D Kyriacou K Lyssiotis T Constantinides V Frangos S. Lymph Node Metastasis and Extrathyroidal Extension in Papillary Thyroid Microcarcinoma in Cyprus: Suspicious Subcentimeter Nodules Should Undergo FNA When Multifocality is Suspected. J Thyroid Res (2020) 2020. 10.1155/2020/3567658 PMC7128046 32351678

[53] Vella V Sciacca L Pandini G Mineo R Squatrito S Vigneri R. The IGF System in Thyroid Cancer: New Concepts. Mol Pathol (2001) 54(3):121. 10.1136/mp.54.3.121 11376121 PMC1187048

[54] Mitsiades N Pazaitou-Panayiotou K Aronis KN Moon H-S Chamberland JP Liu X. Circulating Adiponectin is Inversely Associated With Risk of Thyroid Cancer: In Vivo and In Vitro Studies. J Clin Endocrinol Metab (2011) 96(12):E2023–E8. 10.1210/jc.2010-1908 PMC3232611 21937620

[55] Dossus L Franceschi S Biessy C Navionis AS Travis RC Weiderpass E. Adipokines and Inflammation Markers and Risk of Differentiated Thyroid Carcinoma: The EPIC Study. Int J cancer (2018) 142(7):1332–42. 10.1002/ijc.31172 29168186

[56] McLeod DS Cooper DS Ladenson PW Ain KB Brierley JD Fein HG. Prognosis of Differentiated Thyroid Cancer in Relation to Serum Thyrotropin and Thyroglobulin Antibody Status at Time of Diagnosis. Thyroid (2014) 24(1):35–42. 10.1089/thy.2013.0062 23731273 PMC3887423

[57] Fan YL Li XQ. Expression of Leptin and its Receptor in Thyroid Carcinoma: Distinctive Prognostic Significance in Different Subtypes. Clin endocrinol (2015) 83(2):261–7. 10.1111/cen.12598 25158596

[58] Cheng S-P Yin P-H Hsu Y-C Chang Y-C Huang S-Y Lee J-J. Leptin Enhances Migration of Human Papillary Thyroid Cancer Cells Through the PI3K/AKT and MEK/ERK Signaling Pathways. Oncol Rep (2011) 26(5):1265–71. 10.3892/or.2011.1388 21750869

[59] Uddin S Bavi P Siraj AK Ahmed M Al-Rasheed M Hussain AR. Leptin-R and its Association With PI3K/AKT Signaling Pathway in Papillary Thyroid Carcinoma. Endocrine-related cancer (2010) 17(1):191–202. 10.1677/ERC-09-0153 20008098

[60] Peterson E De P Nuttall R. BMI, Diet and Female Reproductive Factors as Risks for Thyroid Cancer: A Systematic Review. PloS One (2012) 7(1):e29177. 10.1371/journal.pone.0029177 22276106 PMC3261873

[61] Derwahl M Nicula D. Estrogen and its Role in Thyroid Cancer. Endocrine-related Cancer (2014) 21(5):T273–T83. 10.1530/ERC-14-0053 25052473

[62] Freeman EW Sammel MD Lin H Gracia CR. Obesity and Reproductive Hormone Levels in the Transition to Menopause. Menopause (New York NY) (2010) 17(4):718. 10.1097/gme.0b013e3181cec85d PMC2888623 20216473

[63] Lee M Chen G Vlantis AC Tse G Leung B Van Hasselt C. Induction of Thyroid Papillary Carcinoma Cell Proliferation by Estrogen Is Associated With an Altered Expression of Bcl-Xl. Cancer J (2005) 11(2):113–21. 10.1097/00130404-200503000-00006 15969986

[64] Manole D Schildknecht B Gosnell B Adams E Derwahl M. Estrogen Promotes Growth of Human Thyroid Tumor Cells by Different Molecular Mechanisms. J Clin Endocrinol Metab (2001) 86(3):1072–7. 10.1210/jcem.86.3.7283 11238488

[65] Matrone A Ceccarini G Beghini M Ferrari F Gambale C D’Aqui M. Potential Impact of BMI on the Aggressiveness of Presentation and Clinical Outcome of Differentiated Thyroid Cancer. J Clin Endocrinol Metab (2020) 105(4):e1124–e34. 10.1210/clinem/dgz312 31875910

[66] Gąsior-Perczak D Pałyga I Szymonek M Kowalik A Walczyk A Kopczyński J. The Impact of BMI on Clinical Progress, Response to Treatment, and Disease Course in Patients With Differentiated Thyroid Cancer. PloS One (2018) 13(10):e0204668. 10.1371/journal.pone.0204668 30273371 PMC6166948

[67] Nieman KM Romero IL Van Houten B Lengyel E. Adipose Tissue and Adipocytes Support Tumorigenesis and Metastasis. Biochim Biophys Acta (BBA)-Molecular Cell Biol Lipids (2013) 1831(10):1533–41. 10.1016/j.bbalip.2013.02.010 PMC3742583 23500888

[68] Lengyel E Makowski L DiGiovanni J Kolonin MG. Cancer as a Matter of Fat: The Crosstalk Between Adipose Tissue and Tumors. Trends cancer (2018) 4(5):374–84. 10.1016/j.trecan.2018.03.004 PMC5932630 29709261

