# Supplementary material for: Association Between Aggressive Clinicopathologic Features of Papillary Thyroid Carcinoma and Body Mass Index: A Systematic Review and Meta-Analysis
Source: Front Endocrinol (Lausanne). 2021 Jun 30;12:692879. doi: 10.3389/fendo.2021.692879 (PMC8279812; doi:10.3389/fendo.2021.692879)
Supplement: Supplementary file 1 [file DataSheet_1.docx]

**Supplementary File 1.** Funnel plots of the included meta-analyses


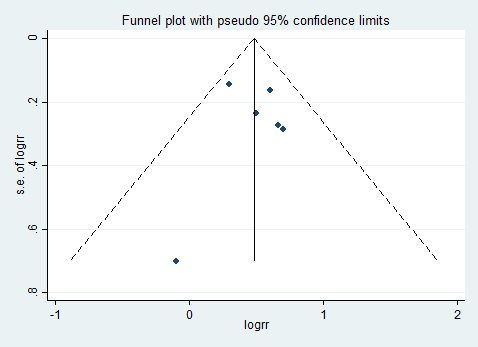


**Supplementary Figure 1.** Funnel plot in the meta-analysis on the association between overweight and TNM Stage.


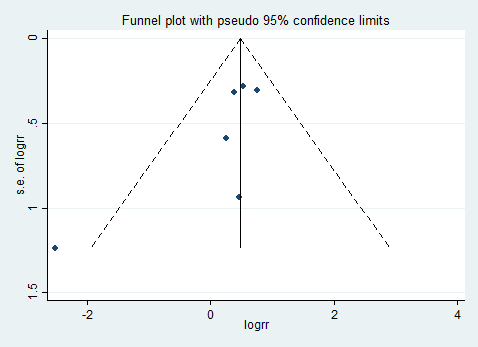


**Supplementary Figure 2.** Funnel plot in the meta-analysis on the association between obesity and TNM Stage.


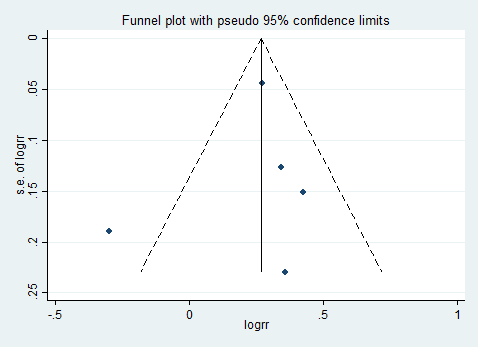


**Supplementary Figure 3.** Funnel plot in the meta-analysis on the association between overweight and tumour size.


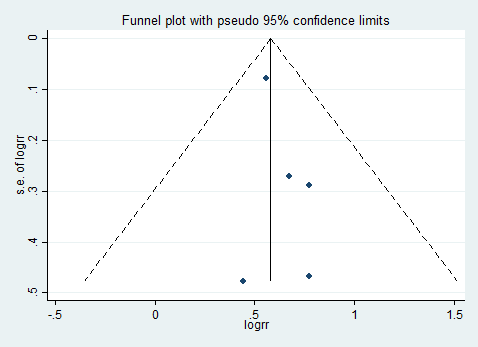


**Supplementary Figure 4.** Funnel plot in the meta-analysis on the association between obesity and tumour size.


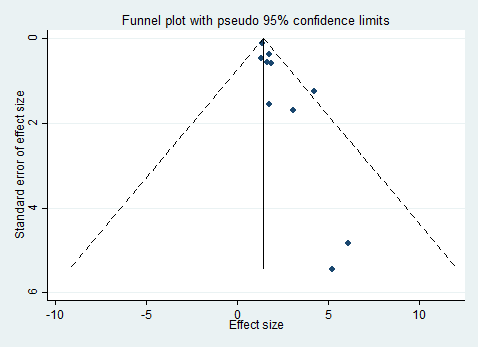


**Supplementary Figure 5.** Funnel plot in the meta-analysis on the association between overweight and Extrathyroidal Extension.


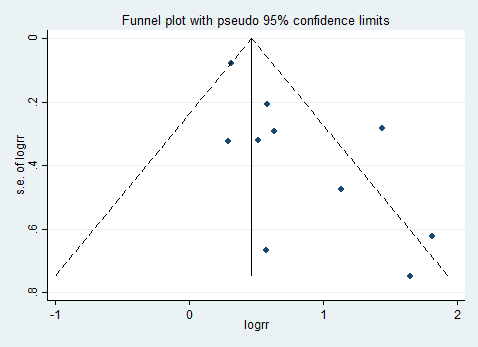


**Supplementary Figure 6.** Funnel plot in the meta-analysis on the association between obesity and Extrathyroidal Extension.


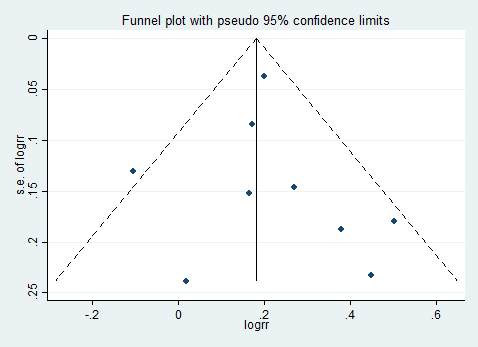


**Supplementary Figure 7.** Funnel plot in the meta-analysis on the association between overweight and Multifocality.


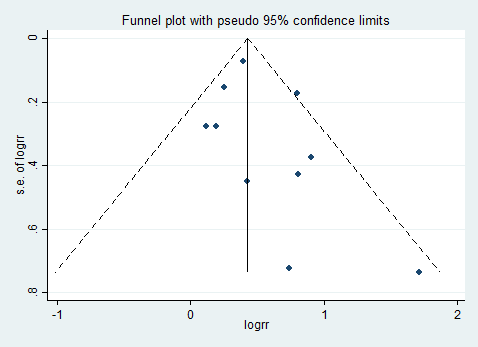


**Supplementary Figure 8.** Funnel plot in the meta-analysis on the association between obesity and Multifocality.


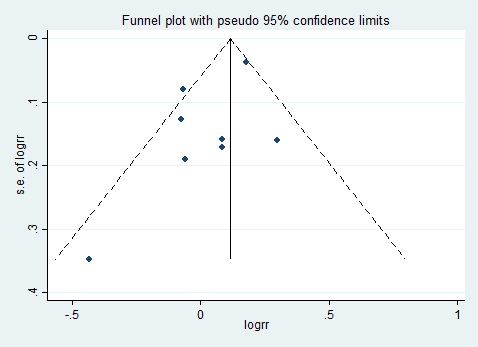


**Supplementary Figure 9.** Funnel plot in the meta-analysis on the association between overweight and LN Metastasis.


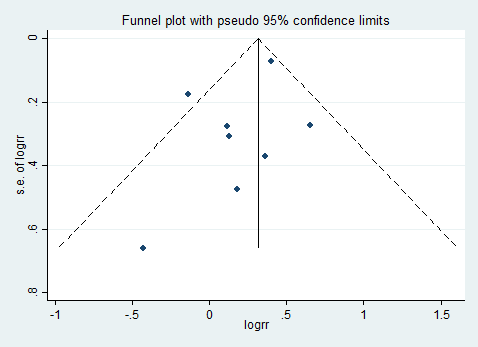


**Supplementary Figure 10.** Funnel plot in the meta-analysis on the association between obesity and LN Metastasis.
