# Supplementary material for: Association Between Aggressive Clinicopathologic Features of Papillary Thyroid Carcinoma and Body Mass Index: A Systematic Review and Meta-Analysis
Source: Front Endocrinol (Lausanne). 2021 Jun 30;12:692879. doi: 10.3389/fendo.2021.692879 (PMC8279812; doi:10.3389/fendo.2021.692879)
Supplement: Supplementary file 3 [file Table_2.docx]

| **KeyWords** | **PubMed**  **(Title/ Abstract)** | **EBSCO**  **(Abstract)** | **Cochrane (Title/ Abstract)** |
| --- | --- | --- | --- |
| ("Papillary thyroid carcinoma") OR ("Papillary microcarcinoma") OR ("PTC") OR ("PTMC") OR ("thyroid carcinoma") OR ("differentiated thyroid carcinoma") OR ("PMCs") OR ("PTMs") OR ("PMC") OR ("Thyroid neoplasm") OR Thyroid Cancer | 81.489 |  |  |
| ("BMI") OR ("Body mass index") OR ("obesity ")OR ("body fat ") OR ("adiposity") OR ("abdominal adiposity") OR ("visceral fat ")OR ("Subcutaneous fat") OR ("Adipose tissue ") OR (" body weight") OR ("overweight") OR ("body composition") OR ("waist circumferences") OR ("hip circumference") OR ("waist to hip ratio") OR ("intra-abdominal fat") | 830.164 |  |  |
| ("lymph nodes") OR ("lymph node") OR ("neoplasm metastasis") OR ("metastasis") OR ("neoplasm metastasis") OR ("neoplasm staging") OR ("TNM staging system") OR ("Staging") OR ("Cancer Staging") OR ("TNM Classification") OR ("Classifications TNM") OR ("Clinicopathologic features") OR ("TNM stage") OR ("ETE") OR ("extrathyroidal extension") OR ("LN metastasis") OR ("lymph node metastasis") OR ("Tumor size") OR ("microscopic invasion") OR ("gross invasion") OR ("distance metastasis") OR ("multifocality") OR ("bilaterality" ) OR ("TNM staging") OR ("AJCC stage") | 633.718 |  |  |
| **#1 AND #2 AND #3** | **250** | **513** | **0** |

**Supplementary Table 2:** Summary of the search strategy
