# Supplementary material for: Association Between Aggressive Clinicopathologic Features of Papillary Thyroid Carcinoma and Body Mass Index: A Systematic Review and Meta-Analysis
Source: Front Endocrinol (Lausanne). 2021 Jun 30;12:692879. doi: 10.3389/fendo.2021.692879 (PMC8279812; doi:10.3389/fendo.2021.692879)
Supplement: Supplementary file 4 [file Table_3.docx]

**Supplementary Table 3.** Different variables used for statistical adjustment by the studies included in the meta-analysis.

| **Advanced tumor-node-metastasis stage** | |
| --- | --- |
| Kim HJ et al., 2013 | Age, gender, thyroid stimulating hormone, total cholesterol, fasting blood sugar |
| Feng JW et al., 2019 | Age, gender, triglycerides, fasting blood sugar |
| Harari A et al., 2012 | Age, gender |
| Liu Z et al., 2015 | Age, gender |
| **Tumor size** | |
| Kim HJ et al., 2013 | Age, gender, thyroid stimulating hormone, total cholesterol, fasting blood sugar |
| Feng JW et al., 2019 | Age, gender, triglycerides, fasting blood sugar |
| Liu Z et al., 2015 | Age, gender |
| Li CL et al., 2020 | Age, WHO-BMI quartiles, thyroid stimulating hormone, free thyroxine |
| **Extrathyroidal extension** | |
| Kim HJ et al., 2013 | Age, gender, thyroid stimulating hormone, total cholesterol, fasting blood sugar |
| Feng JW et al., 2019 | Age, sex, triglycerides, fasting blood sugar |
| Liu Z et al., 2015 | Age, gender |
| Li CL et al., 2020 | Age, WHO-BMI quartiles, thyroid stimulating hormone, free thyroxine |
| Kim SK et al., 2016 | Age, gender, tumor size, bilaterality, extrathyroidal extension, chronic lymphocytic thyroiditis, central lymph node metastasis |
| **Multifocality** | |
| Kim HJ et al., 2013 | Age, gender, thyroid stimulating hormone, total cholesterol, fasting blood sugar |
| Feng JW et al., 2019 | Age, gender, triglycerides, fasting blood sugar |
| Liu Z et al., 2015 | Age, gender |
| Li CL et al., 2020 | Age, WHO-BMI quartiles, thyroid stimulating hormone, free thyroxine |
| Kim SK et al., 2016 | Age, gender, tumor size, bilaterality, extrathyroidal extension, chronic lymphocytic thyroiditis, central lymph node metastasis |
| Kim SH et al., 2015 | Age, lymph node metastasis, lymphatic invasion, BMI |
| **Lymph node metastasis** | |
| Kim HJ et al., 2013 | Age, gender, thyroid stimulating hormone, total cholesterol, fasting blood sugar |
| Feng JW et al., 2019 | Age, gender, triglycerides, fasting blood sugar |
| Liu Z et al., 2015 | Age, gender |
| Li CL et al., 2020 | Age, WHO-BMI quartiles, thyroid stimulating hormone, free thyroxine |
| Kim SK et al., 2016 | Age, gender, tumor size, bilaterality, extrathyroidal extension, chronic lymphocytic thyroiditis, central lymph node metastasis |
| Kim SH et al., 2015 | Age, lymph node metastasis, lymphatic invasion, BMI |
