# Supplementary material for: Association Between Aggressive Clinicopathologic Features of Papillary Thyroid Carcinoma and Body Mass Index: A Systematic Review and Meta-Analysis
Source: Front Endocrinol (Lausanne). 2021 Jun 30;12:692879. doi: 10.3389/fendo.2021.692879 (PMC8279812; doi:10.3389/fendo.2021.692879)
Supplement: Supplementary file 5 [file Table_4.docx]

| **Clinicopathologic Feature** | **P-value for Egger (overweight)** | **P-value for Egger (obesity)** |
| --- | --- | --- |
| Advanced TNM Stage | 0.870 | 0.104 |
| Tumor Size | 0.750 | 0.271 |
| Extrathyroidal Extension | 0.016 | 0.016 |
| Multifocality | 0.951 | 0.260 |
| LN Metastasis | 0.142 | 0.264 |

**Supplementary Table 4**: Results from Egger test
